# Microsatellite instability at a tetranucleotide repeat in type I endometrial carcinoma

**DOI:** 10.1186/1756-9966-27-88

**Published:** 2008-12-31

**Authors:** Yoo Duk Choi, Jin Choi, Jo Heon Kim, Ji Shin Lee, Jae Hyuk Lee, Chan Choi, Ho Sun Choi, Min Cheol Lee, Chang Soo Park, Sang Woo Juhng, Jong Hee Nam

**Affiliations:** 1 Department of Pathology, Chonnam National University Medical School, Gwangju, Republic of Korea; 2 Department of Obstetric and Gynecology, Chonnam National University Medical School, Gwangju, Republic of Korea; 3 Brain Korea 21 Project, Center for Biomedical Human Resources at Chonnam National University, Gwangju, Republic of Korea

## Abstract

**Background:**

Microsatellite instability (MSI) at tri- or tetranucleotide repeat markers (elevated microsatellite alterations at selected tetranucleotide repeat, EMAST) has been recently described. But, the underlying genetic mechanism of EMAST is unclear. This study was to investigate the prevalence of EMAST, in type I endometrial carcinoma, and to determine the correlation between the MSI status and mismatch repair genes (MMR) or p53.

**Methods:**

We examined the 3 mono-, 3 di-, and 6 tetranucleotide repeat markers by PCR in 39 cases of type I endometrial carcinoma and performed the immunohistochemistry of *hMSH2*, *hMLH1*, and p53 protein.

**Results:**

More than two MSI at mono- and dinucleotide repeat markers was noted in 8 cases (MSI-H, 20.5%). MSI, at a tetranucleotide repeat, was detected in 15 cases (EMAST, 38.5%). In remaining 16 cases, any MSI was not observed. (MSS, 42.1%), MSI status was not associated with FIGO stage, grade or depth of invasion. The absence of expression of either one of both *hMSH2 *or *hMLH1 *was noted in seven (87.5%) of eight MSI-H tumors, one (6.3%) of 16 MSS tumors, and five (33.3%) of 15 EMAST tumors. (p = 0.010) The expression of p53 protein was found in one (12.5%) of eight MSI-H tumors, five (31.3%) of 16 MSS tumors, and seven of 15 EMAST tumors. (p = 0.247)

**Conclusion:**

Our results showed that about 38.5% of type I endometrial carcinomas exhibited EMAST, and that EMAST was rarely associated with alteration of *hMSH2 *or *hMLH1*.

## Background

Inherited defects in DNA mismatch repair (MMR) genes, usually *hMLH1 *or *hMSH2*, lead to microsatellite instability (MSI) and subsequent malignancy in hereditary nonpolyposis colon cancer (HNPCC) [1]. While typical of HNPCC cancers, MSI also occurs in a minority of sporadic cancer [2]. Recently, a distinct type of MSI has been described where microsatellite alterations are present at selected tetranucleotide repeat regions; this is referred to as 'elevated microsatellite alterations at selected tetranucleotide repeats' (EMAST) [3-5]. Although the genetic basis for MSI associated with defective MMR is being increasingly clarified, the mechanism underlying microsatellite alterations for EMAST tumors remains to be unclear [3]. Recent work has suggested that EMAST may be associated with alterations in the tumor suppressor gene p53 [4,6].

The incidence of MSI in endometrial carcinoma has recently been described in several articles. It has been shown to be present in 25% to 30% of type I endometrial carcinoma, which is related with estrogenic stimulation, and has been rarely demonstrated in type II endometrial carcinoma, which is unrelated with estrogen [10-12]. However, EMAST in endometrial carcinoma has not yet been studied. The aim of the current study was to evaluate the prevalence and clinicopathologic significance of MSI including EMAST in sporadic type I endometrial carcinoma. We also examined the association between *hMLH1*, *hMSH2 *or p53 and EMAST using immunohistochemical staining techniques.

## Methods

### Sample collection

Formalin-fixed, paraffin-embedded tissue samples from sporadic type I endometrial carcinomas were selected from 39 patients who had undergone hysterectomy at Chonnam National University Hospital during the period from 1998 to 2002. None of these patients met the criteria for a diagnosis of HNPCC. Normal lymph node tissue from the same patient was used for control DNA samples. Hematoxylin and eosin (H&E) -stained slides from each case was reviewed previously for determination of depth of invasion and grade. Histological grade and stage were assigned according to the International-Federation of Obstetrics and Gynecology (FIGO) criteria. The depth of myometrial invasion was divided into no evidence of myometrial invasion, ≤ 50% and > 50%.

### Microsatellite instability assay

#### 1) DNA extraction

Paraffin embedded normal and tumor tissues were cut into 10 § sections. The tumor cells were microdissected under the microscope on the H-E stained slide. One tissue section from each case was transferred with an disposable razor blade to 1 ml of xylene in a 1.5 ml microcentrifuge tube. An equal volume of 100% ethanol was added and the samples were pelleted (10 minutes in microcentrifuge), dried under a vacuum, and digested overnight with TEN buffer (0.1 M NaCl, 10 mM Tris·Cl pH 8.0, 1 mM EDTA pH 8.0) 375 μ*l*, 10% SDS 25 μ*l*, and 20 μg/μ*l *proteinase K 15 μ*l *at 56°. The DNA was isolated by phenol-chloroform extraction and ethanol precipitation.

#### 2) Polymerase Chain Reaction

Each matched pair of normal and tumor DNA was subject to PCR analysis with 12 microsatellite markers. The tetranucelotide repeat markers were MYCL1, D19S394, D9S242, UT5320, D8S321, and L17686. Dinucleotide repeat markers were D2S123, D17S250, and D8S554. Mononucleotide repeat markers were BAT25, BAT 26, and BAT40.

Primer sequences for PCR amplification of microsatellite loci were designed and are shown in Table 1. PCR was performed in a total volume of 20 μ*l *with 10–20 ng DNA, 0.175 uM each primer, 2 mM dNTP, 15 mM MgCl_2 _and 0.5 units of Taq polymerase (Supertaq, Republic of Korea). PCR conditions consisted of 28 to 35 cycles at 92° for 1 min, 53° ~63° for 1 min and 72° for 1 min. The annealing temperature was determined empirically for each primer pair. For microsatellite analysis, one primer was end-labeled with T4 polynucleotide kinase (Takara, Japan) and ^32^P-γ [dATP]. PCR products were separated by electrophoresis in denaturing 7 M urea/6% polyacrylamide gels followed by autoradiography.

**Table 1:** Microsatellite primer for MSI assay

Primer	Primer sequence	AT(°)	PCRcycle	Repeat type
*BAT25*	Forward : 5'-TCGCCTCCA AGAATGTAA GT-3'	55	28	Mono
	Reverse: 5'-TCTGGATTTTAACTATGGCTC-3'			
*BAT26*	Forward : 5'-TGACTACTTTTGACTTCAGCC-3'	55	30	
	Reverse: 5'-GTTTCTAACCATTCAACATTTTTATCCC-3'			
*BAT40*	Forward : 5'-GTAGAGCAAGACCACCTTG-3'	55	30	
	Reverse: 5'-GTTTCTACAACCCTGCTTTTGTTCCT-3'			

*D2S123*	Forward : 5'-AAACAGGATGCCTGCCTTTA-3'	55	35	Di
	Reverse: 5'-GGACTTTCCACCTATGGGAC-3'			
*D17S250*	Forward : 5'-GGAAGAATCAAATAGACAAT-3'	55	35	
	Reverse: 5'-GCTGGCCATATATATATTTAAACC-3'			
*D8S554*	Forward : 5'-TTTCCAGACAGGGCCTA-3'	58	35	
	Reverse: 5'-AATGCACAGGACATAATTT-3'			

*MYCL1*	Forward : 5'-TGGCGAGACTCCATCAAAG-3'	58	35	Tetra
	Reverse: 5'-CCTTTTAAGCTGCAACAATTTTC-3'			
*D19S394*	Forward : 5'-AGACTACAGYGAGCTGTGG-3'	58	35	
	Reverse: 5'-GTGTTCCTAACTACCAGGC-3'			
*D9S242*	Forward : 5'-GTGAGAGTTCCTTCTGGC-3'	56	35	
	Reverse: 5'-ACTCCAGTACAAGACTCTG-3'			
*UT5320*	Forward : 5'-ACCGACAGACTCTTGCCTC-3'	58	35	
	Reverse: 5'-TTGAGATGACCCTGAGACTG-3'			
*D8S321*	Forward : 5'-GATGAAAGAATGATAGATTACAG-3'	58	35	
	Reverse: 5'-ATTCTTCTCATGCCATATCTGC-3'			
*L17686*	Forward : 5'-GCACCAATGCTCCAGAAATG-3'	63	35	
	Reverse: 5'-TCATGGTGCCATGATAGGAG-3'			

AT : Annealing temperature.

Mono : Mononucleotide repeat.

Di : Dinucleotide repeat.

Tetra : Tetranucleotide repeat

#### 3) Criteria for EMAST, MSI, and MSS

EMAST was determined when the new peak, in the tumor samples, appeared in more than one of the tetranucleotide repeat markers, but was not present in the mono or dinucleotide repeat markers. A high frequency of MSI (MSI-H) was present when new peak were observed at two or more (≥ 30–40%) in mono or dinucleotide repeat markers. A low frequency of MSI (MSI-L) was present when the new peak was at one mono or dinucleotide repeat marker. Microsatellite stable (MSS) was present when there was no new peak observed in any of the repeat markers studied.

### Immunohistochemical staining

The immunohistochemical staining in formalin-fixed, paraffin-embedded sections was performed with the labeled avidin-biotin complex peroxidase-AEC (3-amino-9-ethylcarbazol) system. Tissue sections on glass slides were de-paraffinized with xylene, hydrated in serially diluted alcohol, and then immersed in 3% H_2_O_2 _in order to quench endogenous peroxidase activity. Antigen retrieval was performed using citrate buffer (Antigen Retrieval Citra; Biogenex, San Ramon, CA, USA) with a pressure cooker. The sections were then incubated with anti-*hMLH1 *(mouse monoclonal; 1:100 dilution; Zymed, San Francisco, USA), anti-*hMSH2 *(mouse monoclonal; 1:100 dilution; Zymed, San Francisco, USA), and anti-*p53 *(mouse monoclonal; 1:100 dilution; DAKO, Carpinteria, USA). The tissue sections were incubated with horseradish peroxidase (HRP) -conjugated streptavidin and chromogen were developed, and counter-stained with hematoxylin. The staining of tumor nuclei for *hMLH1 *and *hMSH2 *was evaluated and noted as absent or present. For *hMLH1 *and *hMSH2*, loss of nuclear protein expression in tumor cells was considered as a defect in the *hMLH1 *or *hMSH2 *gene. The immunostaining for *p53 *was evaluated within tumor nuclei and divided to absent or present.

### Statistical analysis

We performed statistical analysis using the Fisher's exact test between clinicopathologic status or extent of positive immunoreactivity and the MSS, MSI and EMAST groups. All *p *values represent two-sided statistical tests with statistical significance at *p *< 0.05.

## Results and discussion

### Results

#### CLINICOPATHOLOGIC DATA

The patient age ranged from 30 to 68 (mean 53.4) years. Grade 1 was seen in 20 tumors; grade 2, in 11 tumors; and grade 3, in eight tumors. Most of the patients were FIGO stage I or II (33 of 39; 84.6%), and only six tumors were identified as stage III. In 15 (38.5%) tumors, the tumor was confined to endometrium. A more than 50% myometrial invasion was seen in six tumors (15.4%), and less than 50% in 18 tumors (46.1%). (Table 2)

**Table 2:** Clinicopathologic features, MSI status, and the expression of *hMSH2*, *hMLH1*, and p53 protein in type I endometrial carcinomas

No.					Immunohistochemistry			Tetranucleotide repeat						Mononucleotide repeat			Dinucleotide repeat			Subtype of
	Age	Stage	Grade	Depth	*hMSH2*	*hMLH1*	P53	*MYCL1*	*UT5320*	*D19S394*	*D9S242*	*L17686*	*D8S321*	*BAT25*	*BAT26*	*BAT40*	*D17S250*	*D8S554*	*D2S123*	MSI
1	50	III	2	>50%	+	+	+	H	MSI	H	H	H	H	H	H	H	H	H	H	EMAST
2	67	I	1	<50%	+	+	-	H	H	H	H	H	H	MSI	MSI	H	H	MSI	H	MSI-H
3	56	III	1	<50%	+	+	+	H	H	H	H	H	H	H	H	H	H	H	H	MSS
4	43	I	3	Em	+	+	+	H	H	H	H	H	H	H	H	H	H	H	H	MSS
5	39	II	1	<50%	+	+	-	H	H	H	MSI	H	H	H	H	H	H	H	H	EMAST
6	50	I	1	<50%	+	+	-	H	H	H	H	H	H	H	H	H	H	H	H	MSS
7	54	I	2	<50%	-	+	-	H	H	H	H	H	H	MSI	H	MSI	H	MSI	MSI	MSI-H
8	40	I	2	<50%	-	-	-	H	H	H	H	H	H	MSI	H	MSI	H	H	H	MSI-H
9	51	I	1	Em	+	+	-	H	H	H	H	H	H	H	H	H	H	H	H	MSS
10	55	I	1	<50%	-	+	-	MSI	H	H	H	MSI	H	H	H	H	H	H	H	EMAST
11	60	I	1	<50%	+	+	-	H	H	H	H	H	H	H	H	H	H	H	H	MSS
12	68	III	2	>50%	-	-	-	H	H	H	H	H	H	H	MSI	MSI	H	MSI	H	MSI-H
13	55	I	1	Em	+	+	+	H	MSI	H	H	H	H	H	H	H	H	H	H	EMAST
14	66	I	2	<50%	-	+	-	H	H	H	H	H	H	H	H	H	H	H	H	MSS
15	60	I	2	Em	-	+	-	H	H	H	MSI	H	H	H	H	H	H	H	H	EMAST
16	42	I	1	Em	+	+	-	H	H	H	H	H	H	H	H	H	H	H	H	MSS
17	53	I	2	Em	+	+	-	H	H	H	H	H	H	H	H	H	H	H	H	MSS
18	63	I	3	>50%	-	-	+	H	H	H	H	H	H	MSI	H	MSI	H	MSI	MSI	MSI-H
19	58	I	1	Em	+	+	-	H	H	H	H	H	H	H	H	H	H	H	H	MSS
20	36	I	1	Em	+	+	-	H	H	H	H	H	H	H	H	H	H	H	H	MSS
21	40	III	1	<50%	+	+	-	H	H	H	H	MSI	H	H	H	H	H	H	H	EMAST
22	68	I	1	<50%	+	-	-	H	H	H	H	H	H	MSI	MSI	MSI	H	H	H	MSI-H
23	41	I	2	Em	+	+	+	H	H	H	H	MSI	H	H	H	H	H	H	H	EMAST
24	50	I	2	<50%	+	+	-	H	H	H	H	H	H	H	H	H	H	H	H	MSS
25	54	I	1	Em	+	+	-	H	MSI	H	H	MSI	H	H	H	H	H	H	H	EMAST
26	51	I	3	<50%	+	-	-	MSI	MSI	MSI	H	H	H	H	H	H	H	H	H	EMAST
27	63	I	3	Em	+	+	+	H	H	H	H	H	H	H	H	H	H	H	H	MSS
28	46	I	3	Em	+	+	+	H	H	H	H	H	H	H	H	H	H	H	H	MSS
29	64	III	3	>50%	+	+	+	H	H	H	H	MSI	H	H	H	H	H	H	H	EMAST
30	44	II	3	Em	+	+	+	H	H	H	MSI	H	H	H	H	H	H	H	H	EMAST
31	55	III	3	>50%	-	+	+	H	H	H	H	MSI	H	H	H	H	H	H	H	EMAST
32	44	I	1	<50%	-	-	-	H	H	H	H	H	H	MSI	MSI	MSI	MSI	H	MSI	MSI-H
33	43	I	1	<50%	+	+	-	H	H	H	MSI	H	H	H	H	H	H	H	H	EMAST
34	55	I	1	<50%	+	+	+	H	H	H	H	H	H	H	H	H	H	H	H	MSS
35	51	I	1	>50%	+	+	-	H	H	H	H	H	H	H	H	H	H	H	H	MSS
36	33	I	2	Em	+	+	-	H	H	H	H	H	H	H	H	H	H	H	H	MSS
37	49	I	1	Em	+	-	-	MSI	H	H	MSI	H	H	H	H	H	H	H	H	EMAST
38	52	I	1	<50%	+	+	+	H	H	H	H	H	MSI	H	H	H	H	H	H	EMAST
39	30	I	2	<50%	+	-	-	H	H	H	H	H	H	H	MSI	MSI	MSI	MSI	MSI	MSI-H

Em : Endometrium H: Heterozygosity.

MSS : Microsatellite stable.

MSI : Microsatellite instability.

MSI-H : Microsatellite instability – high frequency.

EMAST : Elevated microsatellite alterations at selected tetranucleotide repeat.

#### Molecular data

**Figure 1:**
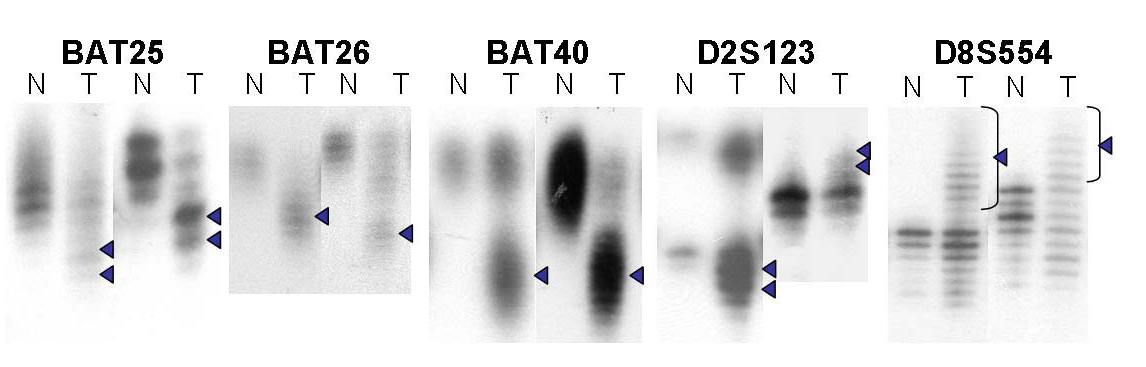
**MSI at mono- and dinucleotide repeat markers in type I endometrial carcinioma**. Alterations in the electrophoretic mobility of PCR products from tumor compared with normal tissue DNA are seen (arrowheads). (N: normal, T: tumor).

**Figure 2:**
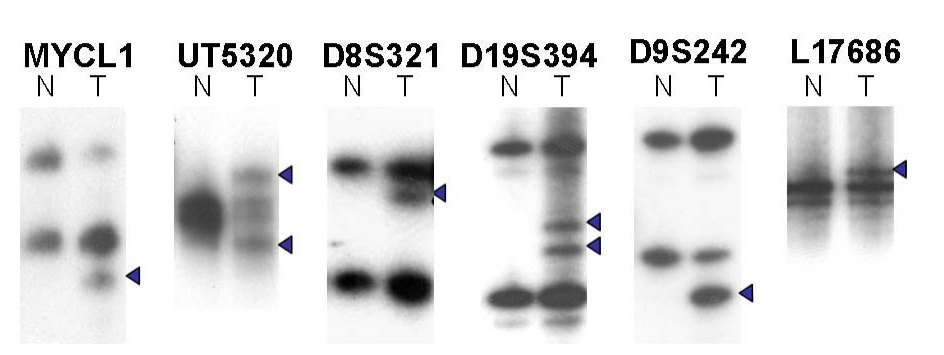
**MSI at tetranucleotide repeat markers (EMAST) in type I endometrial carcinioma**. Alterations in the electrophoretic mobility of PCR products from tumor compared with normal tissue DNA are seen (arrowheads). (N: normal, T: tumor).

Successful PCR amplification of six mono- or dinucleotide repeat markers and six tetranucleotide markers was obtained for 39 tumors and each normal lymph node samples. The data is shown in Table 2. Microsatellite instability at mono- or dinucleotide repeat markers was noted in eight of 39 tumors (20.5%). All eight tumors displayed MSI at two or more loci for mono and dinucleotide repeat markers. Therefore, these eight tumors were categorized as the MSI-H group. There were no tumors classified as MSI-L. The MSI rates at mono or dinucleotide repeat markers were as follows: 15.4% (6/39) in BAT25, 12.8% (5/39) in BAT26, 17.9% (7/39) in BAT40, 5.1% (2/39) in D17S250, 12.8% (5/39) in D8S554, and 10.3% (4/39) in D2S123. (Figure 1) Microsatellite instability at tetranucleotide repeat markers was observed in 15 of 39 tumors (38.5%). All 15 tumors did not exhibit any MSI at the mono- or dinucleotide repeat markers, and were categorized as EMAST. Among 15 EMAST tumors, 11 tumors displayed MSI at one locus for tetranucleotide repeat markers, three displayed at two loci, and one displayed at three loci. The highest MSI rates, for tetranucleotide repeat markers, were seen in *L17686 *(7/39, 17.9%), followed by *D9S242 *(4/39, 10.3%), *UT5320 *(4/39, 10.3%), *MYCL1 *(3/39, 7.7%), *D19S394 *(1/39, 2.6%), and *D8S321 *(1/39, 2.6%). (Figure 2) MSI was not observed in 16 of 39 tumors (41.0%) for all markers studied. Therefore, these 16 tumors were categorized as MSS. There was no significant differences between MSI-H, EMAST and MSS group in respect to stage (*p *= 0.28), grade (*p *= 0.66) and depth of invasion (*p *= 0.06). (Table 3)

**Table 3:** MSI status in relation to clinicopathologic features.

		No.	MSS	MSI-H	EMAST	*p*-value
Stage	I or II	33	15(93.8%)	7(78.5%)	11(73.3%)	0.28
	III	6	1(6.2%)	1(12.5%)	4(26.7%)	

Grade	1	20	9(56.3%)	3(37.5%)	8(53.3%)	0.66
	2	11	4(25.0%)	4(50.0%)	3(20.0%)	
	3	8	3(18.7%)	1(12.5%)	4(26.7%)	

Depth	Endometrium	15	9(56.3%)	0(0.0%)	6(40.0%)	0.06
	<50% of myometrium	18	6(37.5%)	6(75.0%)	6(40.0%)	
	>50% of myometrium	6	1(6.2%)	2(25.0%)	3(20.0%)	

Total		39	16(41.0%)	8(20.5%)	15(38.5%)	

MSS : Microsatellite stable.

MSI : Microsatellite instability – high frequency.

EMAST : Elevated microsatellite alterations at selected tetranucleotide repeat.

#### Immunohistochemical staining data

##### 1) hMSH2 and hMLH1

**Figure 3:**
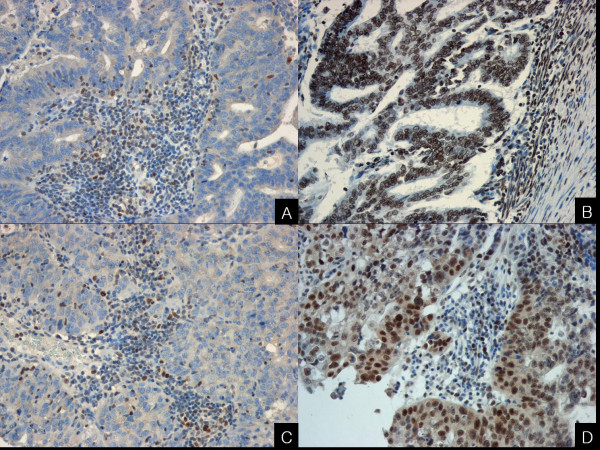
**Examples of type I endometrial carcinoma with (A) loss of *hMSH2 *protein expression, (B) preservation of *hMSH2 *protein expression, (C) loss of *hMLH1 *protein expression, and (D) preservation of *hMLH1 *protein expression**. Positive expression for *hMSH2 *or *hMSH1 *is noted in non-tumor cells. (A-D, X200).

Loss of nuclear *hMSH2 *protein expression was seen in 23.0% (9 of 39), and loss of *hMLH1 *in 20.5% of tumors (8 of 39). In 13 of total 39 tumors (33.3%), the protein expression loss was found at least one of either *hMSH2 *or *hMLH1*. (Figure 3) No correlation between the loss of *hMSH2 *or *hMLH1 *and clinicopathologic data was observed. Loss of *hMSH2 *or *hMLH1 *was mainly seen in MSI-H group (7 of 8), and was rarely seen in MSS group (1 of16). The MSI-H group was significantly correlated with loss of at least one of both proteins. (p < 0.01) In 33.3% (5 of 15) of EMAST group, the loss of *hMSH2 *or *hMLH1 *was observed. (Table 4) Of 11 tumors that displayed MSI at one locus for tetranucleotide markers, nine tumors showed positive immunoreactivity for both *hMSH2 *and *hMLH1*. Of four tumors that displayed MSI at two or three loci for tetranucleotide repeat markers, three tumors showed the expression loss of at least one of either *hMSH2 *or *hMLH1*.

**Table 4:** MSI status in relation to the expression of *hMSH2*, *hMLH1*, and p53 protein

	IHC	No.	MSS	MSI-H	EMAST	*p*-value
*hMSH2*	Positive	30	15(92.8%)	3(37.5%)	12(80.0%)	0.01
	Negative	9	1(7.2%)	5(62.5%)	3(20.0%)	

*hMLH1*	Positive	31	16(100.0%)	2(33.3%)	13(86.7%)	<0.01
	Negative	8	0(0.0%)	6(66.7%)	2(13.3%)	

*hMSH2 *and *hMLH1*	Positive, both	26	15(93.7%)	1(12.5%)	10(67.7%)	<0.01
	Negative, at least one	13	1(6.3%)	7(87.5%)	5(33.3%)	

p53	+	13	5(31.3%)	1(12.5%)	7(46.7%)	0.25
	-	26	11(68.7%)	7(87.5%)	8(53.3%)	

Total		39	16	8	15	

IHC : Immunohistochemistry.

MSS : Microsatellite stable.

MSI : Microsatellite instability – high frequency.

EMAST : Elevated microsatellite alterations at selected tetranucleotide repeat

##### 2) p53

The p53 immunohistochemical expression was noted in 31.3% (5 of 16) of MSS, 12.5% (1 of 8) of MSI-H, and 46.7% (7/15) of EMAST. Although p53 expression was more common in the EMAST group than the other groups, the difference was not statistically significant. (p = 0.25) (Table IV). The p53 expression was noted in seven of 11 tumors that displayed MSI at one locus for tetranucleotide markers (63.6%). None of four tumors displayed MSI at two or three loci for tetranucleotide markers showed the p53 expression. For the clinicopathologic data, p53 expression was not correlated with stage and depth of invasion. But, observation of p53 expression was noted in 87.5% (7/8) of grade 3, 18.2% (2/11) of grade 2, and 20.0% (4/20) of grade 1 tumors. Thus, p53 expression was highly associated with FIGO grade 3 tumors. (p < 0.01)

## Discussion

In our study, the incidence of EMAST in type I endomterial carcinoma was 38.5%. To date, those tumors showing the highest levels of EMAST are associated with known environmental carcinogens, for example, cigarette smoking or sunlight exposure, suggesting a causal relationship [13]. For example, EMAST is common in head and neck (56%), lung (51%) and bladder cancer (21–40%), but infrequently found in renal (12%) and prostate cancer (6%) [5,14]. Danaee *et al*. found 75.4% and 43.3% of MSI at tetranucleotide repeat markers in non-melanomatous skin cancer and bladder tumors, respectively [14]. Wooster *et al*. reported that human cancers including breast (11%), brain (2%), ovarian (10%) cancers and soft tissue sarcoma (11%) exhibited EMAST [15]. The EMAST rate in type I endometrial cancer was relatively higher than that of kidney, prostate, brain and ovary. The estrogen which is widely known as a causal factor in type I endometrial carcinoma can be possible as a novel factor associated with EMAST. However, the tetranucleotide repeat makers are different between our study and others. In order to clarify whether the estrogen is associated with EMAST or not, a standard gene panel for EMAST study, like Bethesda panel used in mono- & dinucleotide repeat markers [3], should be requested.

Seven of eight MSI-H tumors (87.5%) of our study showed a loss of at least one of both *hMLH1 *and *hMSH2*. However, for the EMAST group, it was observed in five of 15 tumors (33.3%). Malfunction of the MMR genes including *hMLH1 *and *hMSH2 *can result from a somatic mutation, deletion, and hypermethylation of the coding region. In sporadic endometrial carcinoma cell lines, somatic mutations are infrequent, and hypermethylation of the *hMLH1 *promoter region appears to be the main cause of gene silencing [21,22]. Thus, our results said that a defect of *hMLH1 *or *hMSH2 *was less frequently detected in EMAST group than MSI-H group.

The majority of sporadic endometrial carcinomas (at least approximately 70%–80%), are designated as type I endometrial carcinomas and are estrogen related. Histologically, most type I endometrial carcinomas show an endometrioid differentiation and are of low grade. About 10–20% of endometrial carcinomas, designated as type II endometrial carcinomas, are estrogen-unrelated; these tumors usually present in older patients and are typically high-grade carcinomas, with non-endometrioid differentiation [8]. Molecular data from multiple studies support the hypothesis of different genetic pathways for the development of type I and type II endometrial carcinoma. The most frequent alteration in type 1 endometrial carcinoma is the *PTEN *inactivation by mutation, followed by MSI and mutations of *K-ras *and *β-catenin*. In type II endometrial carcinoma, alterations of p53 and loss of heterozygosity (LOH), at several chromosomal loci, are thought to drive the process of neoplastic transformation [8,9]. In this study, the incidence of MSI, in type I endometrial carcinoma, is similar to that previously reported [10,12]. Our study for type I endometrial carcinomas showed no significant differences between three groups with regard to stage, FIGO grade, and the extent of myometrial invasion. Peiro et al. reported that tumors in an advanced FIGO stage (III to IV) were more frequently MSI positive than those in a low stage (I to II) [20]. This difference can be interpretated as two problems : 1) Peiro's study included type II endometrial carcinomas as well as type I endometrial carcinomas. 2) The samples included in our study are not evenly distributed in aspect of FIGO stage. Most of the samples were FIGO stage I or II (33 of 39; 84.6%). The unbalanced stage of our study' cases seems to be a factor affecting the result.

Alteration of the p53 gene is a common event in many cancers, and is primarily associated with protein overexpression. Given its role in maintaining genomic stability, it is possible that altered p53 might play a mechanistic role in the genesis of EMAST [14]. In fact, a recent report by Ahrendt et al. found that most tumors with EMAST also had p53 gene mutations [4]. In this study, although the p53 gene was expressed more in EMAST (+) tumors than EMAST (-) tumors, this difference was not statistically significant. The p53 mutation was known as a genetic alteration in type II endometrial carcinomas [24]. However, p53 mutation was also found in a subset of approximately 10–20% of type I endometrial carcinomas, which were mostly grade 3 tumors [25]. Catasus et al. found that most grade 3 tumors in type I endometrial carcinomas showed the immunohistochemical expression for p53 [10], this finding is presented in our result. The p53 protein was highly expressed in grade 3 tumors (p < 0.001).

Among tetranucleotide repeat markers that were examined in our study, MSI was highly detected in *L17686 *gene (7/39). The gene is located in long arm of chromosome 7, and was also highly detected marker in lung cancer and head & neck cancer [17]. *UT5320 *and *MYCL1 *that showed the higher detection rate in bladder cancer were observed in 10.3% (4/39) and 7.7% (3/39) in type I endometrial carcinomas of our study, respectively [18,19]. The detection rate at each tetranucleotide repeat maker seems to be different according to organ or tumor types. In order to solve this question, EMAST study within more various organ and tumor types should be preceded.

To date, seven human MMR genes whose products function in MMR have been identified. These include homologs of the *Escherichia coli mutS *– *hMSH2, hMSH3, hMSH6 *-, and *mutL *homologs – *hMLH1, hPMS2, hMLH3 *and *hPMS1 *[26,27]. DNA strand slippage at tri- or tetranucleotide repeats during replication will result in formation of loops containing more than three extra nucleotides. A complex between the heterodimer MutLα (*hMLH1/hPMS2*) and the heterodimer MutSβ (*hMSH2/hMSH3*) is responsible for repairing these loops [28]. Recently, yeast MLH3 was found to form a complex with y *MLH1 *and to participate in repairing some groups of loops in collaboration with MutSβ. This suggests that *hMLH3*, a homolog of yeast *MLH3*, might have a similar function. Therefore, the loss of the *hMSH3 *and/or *hMLH3 *function may result in loss of loop repair, and eventually may be responsible for EMAST. This should inspire further investigations to prove a correlation between *hMSH3 *and/or *hMLH3 *and EMAST

In this study, 11 tumors of 15 EMAST (73.3%) showed MSI at one locus for tetranucleotide repeats, and four tumors (26.7%) showed MSI at two or more loci. Of 11 tumors, a defect of *hMLH1 *or *hMSH2 *expression was found in 18.2% (2 of 11) and p53 overexpression was observed in 63.6% (7 of 11). In 4 tumors at two or more loci, 3 tumors of 4 showed the expression loss of *hMLH1 *or *hMSH2 *and none showed the p53 expression. It is possible to be a different genetic alteration between two groups. However, after the molecular events associated with EMAST become clear, the research about the genetic difference appears to be a subject.

In conclusion, the incidence of EMAST in endometrioid carcinoma was 38.5%, and an alteration of *hMSH2 *or *hMLH1 *was not frequent in EMAST.

## Abbreviations

MMR: Mismatch repair; MSI: Microsatellite instability; HNPCC: Hereditary non-polyposis colon cancer; PCR: Polymerase chain reaction; EMAST: Elevated microsatellite alterations at selected tetranucleotide repeats; H&E: Hematoxylin and eosin; FIGO: International-Federation of Obstetrics and Gynecology; MSI-H: a high frequency of MSI; MSI-L: A low frequency of MSI; MSS: Microsatellite stable; HRP: Horseradish peroxidase.

## Competing interests

The authors declare that they have no competing interests.

## Authors' contributions

YDC, JSL, and JHN carried out the microsatellite instability assay and participated in the design of the study. JC and HSC collected the samples and drafted the manuscript. CSP and JHK carried out the immmunohistochemical staining. CC performed the statistical analysis. JHL, MCL and SWJ conceived of the study, and participated in its design and coordination and helped to draft the manuscript. All authors read and approved the final manuscript.
